# Pre-harvest dynamics of *Aspergillus* section *Flavi* and aflatoxin risk in hazelnut orchards of Azerbaijan

**DOI:** 10.3389/fpls.2026.1791562

**Published:** 2026-03-05

**Authors:** Alessia Casu, Marco Camardo Leggieri, Giorgio Chiusa, Eugenio Zagottis, Giuseppe Genova, Paola Battilani

**Affiliations:** 1 Department of Sustainable Crop Production, Università Cattolica del Sacro Cuore, Piacenza, Italy; 2 Soremartec Italia S.r.l, Piazzale Pietro Ferrero 1, Alba, Italy

**Keywords:** *Aspergillus flavus*, Azerbaijan, food safety, microclimate, mycotoxins, pre-harvest infection, tree nut crops

## Abstract

Hazelnuts (*Corylus avellana L*.) are susceptible to aflatoxin contamination, with recurrent notifications reported in Azerbaijan. A three-year study (2023–2025) was conducted in 30 orchards across three main hazelnut-producing districts to investigate the pre-harvest dynamics of *Aspergillus* section *Flavi*. Fungal populations were monitored at four phenological stages (BBCH 70–89), and aflatoxin levels were assessed at harvest and late-harvest. Fungal abundance and incidence were significantly affected by geographical area, year, and growth stage (*P* < 0.01). *A*. section *Flavi* populations were higher in warmer districts such as Qabala and Zaqatala (up to 1.7 Log_10_ CFU/g) compared with Khachmaz (1.0 Log_10_ CFU/g), with relative incidence reaching 13.8% in Zaqatala. Fungal abundance peaked during nut filling (BBCH 79), reaching 2.4 Log_10_ CFU/g, with a corresponding incidence of 18.5%. Marked interannual variability was observed, with *A*. section *Flavi* incidence increasing from 1.2% in 2023 to 40.8% in 2024. Despite the frequent isolation of *A*. section *Flavi*, aflatoxin levels in standard orchard samples collected at BBCH 89 remained generally below EU limits (≤5 μg/kg). In contrast, late-harvest samples showed markedly higher contamination, with total aflatoxin concentrations ranging from 71.2 to 752.8 μg/kg, particularly in ground-collected nuts. These findings indicate that pre-harvest aflatoxin risk is primarily driven by orchard microclimate and harvest timing, highlighting the importance of Good Agricultural Practices, timely harvest, and rapid post-harvest drying.

## 1 Introduction

Hazelnut (*Corylus avellana* L.) is a versatile and economically important crop, widely consumed directly and extensively used in the confectionery and chocolate industry. Azerbaijan has a long-standing tradition of hazelnut cultivation, where the crop holds a key role both for domestic supply and international trade. In 2023, the country ranked as the fourth-largest global producer (FAO, 2024; FAOSTAT, 2024). Despite the expansion of intensive orchards in recent years, hazelnut production in Azerbaijan remains largely dominated by smallholder farms with limited mechanization, which constrains the sector’s full potential.

Hazelnuts are susceptible to infection by several microorganisms, some of which cause spoilage or produce secondary metabolites that threaten food safety. Among them, fungi belonging to *Aspergillus* section *Flavi* are of major concern due to their ability to synthesize aflatoxins (AF), a group of highly toxic and carcinogenic metabolites (Salvatore et al., 2023). Aflatoxin B_1_ (AFB_1_) is classified as a Group 1 carcinogen by the International Agency for Research on Cancer (IARC, 1993). In the European Union, Commission Regulation (EU) No 2023/915 establishes strict maximum limits for AF occurrence in hazelnuts, with maximum content of 5 µg/kg for AFB_1_ and 10 µg/kg for total AF for nuts intended for direct consumption, and 8 µg/kg and 15 µg/kg respectively for nuts subjected to sorting or processing before consumption. Exceeding these thresholds not only poses severe health risks but also leads to product rejection, economic losses, and trade restrictions (Bayman et al., 2002; Donner et al., 2015; European Commission (EC), 2023; Molyneux et al., 2007).

The European Commission Rapid Alert System for Food and Feed (RASFF) has consistently reported notifications for AF contamination in hazelnuts. In recent years, a significant proportion of these alerts originated from hazelnut batches produced in Azerbaijan, underlining the growing relevance of this issue for their national hazelnut production (Owolabi et al., 2023; RASFF, 2025).

Previous studies have shown that fungal occurrence and AF production may occur at different stages of the hazelnut production chain, including in the orchard, at harvest, and during post-harvest handling and storage (Ozay et al., 2008). Although AF levels detected directly in orchards were generally low, inadequate drying and storage conditions can substantially exacerbate contamination (Marín and Ramos, 2016; Casu et al., 2026). Importantly, the onset of infection in the field remains poorly understood compared to other crops, such as maize, peanuts or pistachios, where pre-harvest colonization by *A. flavus* is well documented (Angle et al., 1989; Battilani et al., 2013; Cole et al., 1986; Crosta et al., 2024; Doster and Michailides, 1994a; Doster and Michailides 1994b; Doster and Michailides, 1995; Horn, 2005; Jones, 1980; Kaminiaris et al., 2020; Payne and Widstrom, 1992).

Agricultural soils represent a major reservoir of *Aspergillus* species, with *A. flavus* often dominating the soil mycobiota of hazelnut orchards (Şen and Kabak, 2025). Spores can spread through organic debris, soil particles, or insect vectors, while kernel susceptibility is strongly influenced by shell integrity, often compromised by insect damage. Surveys across different producing Azerbaijani districts have shown that, although *Penicillium* and *Aspergillus* are consistently among the most frequent genera associated with hazelnuts, the proportion of toxigenic isolates and their AF-producing capacity can vary considerably (Lombardi et al., 2022; Ortega et al., 2020). This highlights that AF risk results from a combination of host, fungal, and environmental factors, and that infection may already start in the orchard under favorable conditions.

The aim of this study was to investigate the infection dynamics of *A. flavus* in Azerbaijani hazelnut orchards, focusing on four key developmental stages, from early inflorescence to fully mature kernels. By monitoring the crop across these growth stages, the study aimed to identify the timing of initial fungal colonization and to determine when hazelnuts are most susceptible to pathogen attack. In addition, the study assessed whether AF contamination can already occur under field conditions, before harvest.

## 2 Materials and methods

### 2.1 Orchards’ location

**Figure 1:**
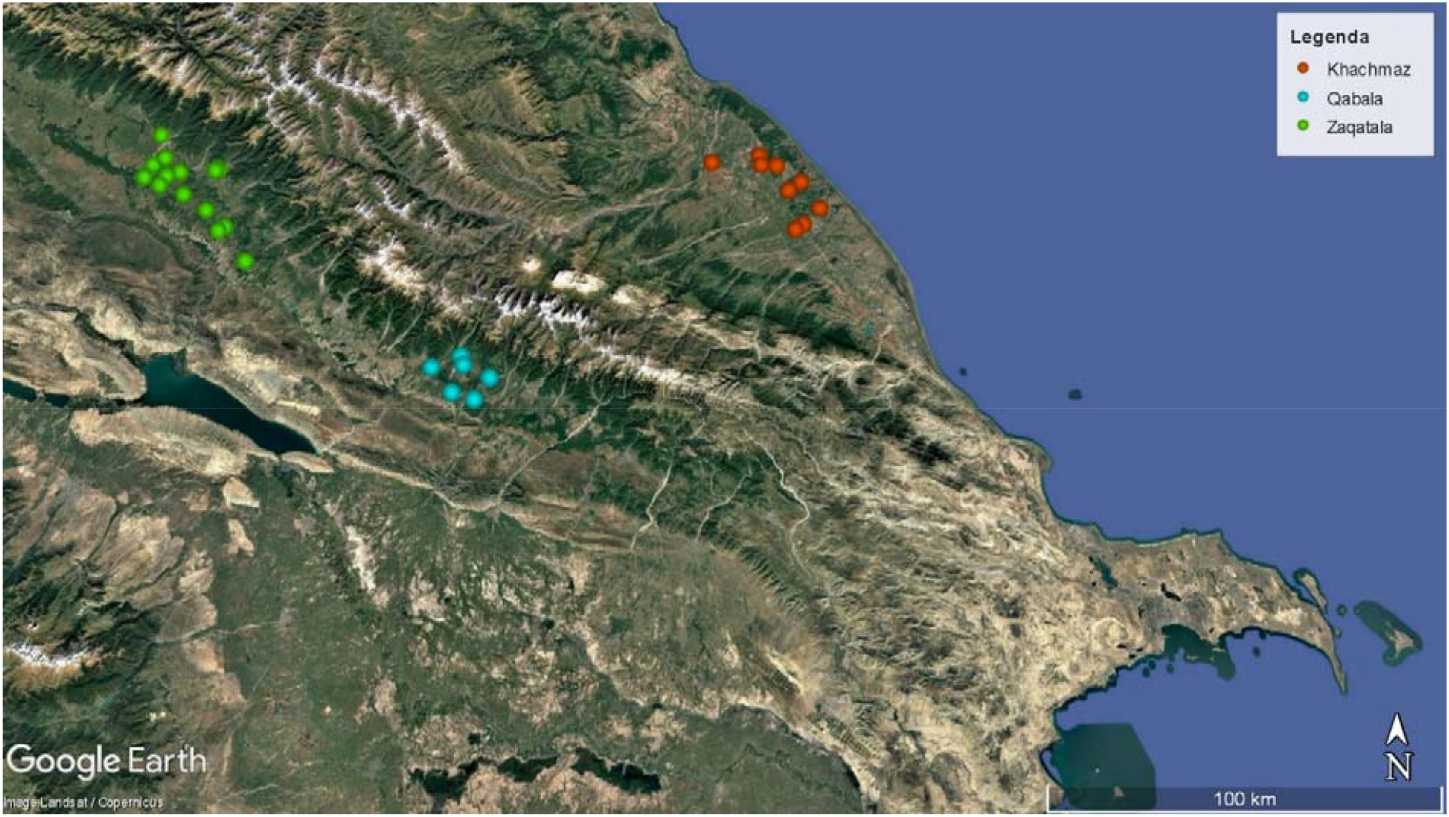
Hazelnut orchards sampled from 2023–2025 in the territory of Azerbaijan (orange for Khachmaz, light blue for Qabala, green for Zaqatala). Map created using Google Earth (© Google; data providers as indicated on the map), accessed January 2026.

Thirty hazelnut orchards located in Azerbaijan were selected at the beginning of the study and were followed for three cropping seasons (2023 to 2025). Sixteen orchards were in Zaqatala, indicated as the main productive area of Azerbaijan, while eight orchards were selected in the Khachmaz and six in the Qabala districts (**Figure 1**; **Supplementary Table 1**).

The orchards represented small-scale farming systems with diverse tree ages, agronomic practices, and management approaches. Most sites were managed under organic or low-input conditions, generally without the use of synthetic fertilizers or pesticides. For illustrative purposes, orchard Zrc n39 relied exclusively on manure and occasional irrigation. More detailed agronomic information was available for orchard Kagr n10, which was managed with a conventional system including applications of ammonium phosphate, manure, urea, Bordeaux mixture, and submersion irrigation; however, this level of detail was not consistently available for the remaining orchards.

Two main cultivars were represented in the study: *Ata-baba*, collected in Zaqatala and Qabala orchards, and *Khachmaz*, sampled in the Khachmaz district.

### 2.2 Meteorological data

Following the study by Battilani et al. (2018), weather data were collected through MeteoBlue (MeteoBlue, 2025) for each district of the study (GPS coordinates for Khachmaz district: N 41.606 and E 48.627, Qabala district: N 40.934 and E 47.790, Zaqatala district: N 41.632 and E 46.645). Hourly data of air temperature (T, °C), air relative humidity (RH, %) and rainfall (R, mm) were recorded from 1^st^ January to 31^st^ December for each of the 3 years included in the study.

Four different time periods were defined (P1: 1 January – 30 April; P2: 1 May – 30 June; P3: 1 July – 31 July; P4: 1 August – 30 August) and for each period six parameters were calculated: mean air temperature (Tm, °C), Degree Day (DD, °C), summation of DD (ΣDD, °C), total rainfall (R, mm), summation of R (ΣR, mm) and mean air relative humidity (RHm, %).

Differences in meteorological variables were assessed using one-way analysis of variance (ANOVA) performed in R (R Foundation for Statistical Computing, Vienna, Austria), testing the effects of time period, geographical area and year.

### 2.3 Hazelnut sampling design

Four hazelnut growth stages were monitored: BBCH 70 (ovary enlargement; occurring in April), BBCH 71 (beginning of fruiting; May), BBCH 79 (immature fruit; June-July), and BBCH 89 (ripe fruit; August-September) (Taghavi et al., 2022). For BBCH 70 and BBCH 71, approximately 1000 clusters per orchard were collected (corresponding to ~3000 fruits at maturity), while for BBCH 79 and BBCH 89 a total of 3000 in-shell nuts were harvested.

The number of trees sampled per orchard was specifically defined for this study based on orchard surface area, with the aim of ensuring a representative coverage of each site while accounting for orchard heterogeneity. This approach allowed proportional sampling across orchards of different sizes. In particular, 4 trees were sampled for orchards of 0.5 ha, 6–8 trees for orchards of 0.5–1 ha, 10–12 trees for orchards of 1-1.5 ha. Trees were selected within the orchard, avoiding the first two rows on the edge of the field and the sampling was conducted following a diagonal pattern. Hazelnuts were harvested at different heights from the ground (basal, middle and apical portion), on different branches, following the four cardinal points. Although planting density information was not consistently available, the sampling design focused on spatial representativeness and on maximizing the number of biological units collected per orchard, which is considered critical when dealing with point-source contamination.

After collection, samples were dried in a warm, well-aired space. Subsequently, samples were stored at 18 °C in paper or fabric bags and analyzed shortly after collection (maximum 5 days after delivery).

During the 2025 season, six supplementary late-season samples were collected in Zaqatala district to evaluate the effect of delayed harvesting on fungal occurrence and AF contamination. The samples corresponded to three orchards, each represented by two paired lots, one collected directly from the tree and one from the ground, to reproduce typical late-harvest conditions observed in commercial settings. One of these orchard pairs (tree and ground) originated from a site monitored during the study (ZRC n39), while the remaining two pairs were obtained from independent orchards located in Zaqatala district, where harvesting was still ongoing in mid-September. All late-harvest samples were collected approximately three weeks after the standard BBCH 89 stage.

### 2.4 Fungi quantification and morphological characterization

For the first two stages (BBCH 70 and BBCH 71), the entire set of collected material (1000 clusters per orchard) was processed, whereas for BBCH 79 and BBCH 89 a representative subsample of 300 hazelnuts per orchard, randomly selected after accurate mixing, was analyzed. Nuts from the latter two stages were shelled and ground using a household grinder (Moulinex, Groupe SEB, Ecully, France).

Microbiological analysis was conducted by determining colony-forming units per gram (CFU/g) of inflorescences or kernel flour. One gram of homogenized sample was suspended in 9 mL of 0.1% peptone water, vortexed (ZX3, Genelab Srl, Perugia, Italy), and serially diluted (10^-1^ to 10^-3^). Aliquots of 500 µL were spread in five replicates on Dichloran Rose Bengal Chloramphenicol Agar (DRBC; Biolife Italiana S.r.l., Monza, Italy) supplemented with 0.05 g/L chloramphenicol and incubated at 31 °C in darkness for three days (Cotty, 1994; Jaime-Garcia and Cotty, 2007; Casu et al., 2026).

After incubation, colonies were preliminarily assigned to major fungal groups based on macroscopic and microscopic characteristics, namely *Alternaria* spp., *Aspergillus* section *Flavi*, *Aspergillus* section *Nigri*, *Fusarium* spp., and *Penicillium* spp., following standard morphological keys (Pitt and Hocking, 1997; Leslie and Summerell, 2008; Lawrence et al., 2016). Colonies belonging to each group were counted separately on each plate. For each dilution, only plates within the countable range were used to estimate fungal load. Counts were converted to CFU/g according to the formula CFU/g = (mean colony count × dilution factor × 2), where 2 accounts for plating 0.5 mL of the initial 1:10 homogenate.

Colonies presumptively assigned to *A.* section *Flavi* were further subcultured on 5/2 agar medium (5% V8 juice, 2% agar, pH 5.2) and incubated at 31 °C for 5–7 days. Species/section-group identification was then refined based on macroscopic features (colony color, texture, margins, reverse pigmentation, and presence of sclerotia) and confirmed by microscopic examination of wet mounts in sterile water, focusing on unbranched conidiophores terminating in globose vesicles and bearing chains of rough-walled conidia, which are characteristic of the genus *Aspergillus*. Detailed micrometric measurements and species-level differentiation were not performed, as molecular methods were later applied for definitive species identification (Jaime-Garcia and Cotty, 2007; Samson et al., 2014; Raper and Fennell, 1965).

### 2.5 Molecular confirmation of *A.* section *Flavi* isolates

To confirm species identity, representative isolates were selected according to the predefined study design. Specifically, for each geographical area, one isolate per growth stage was analyzed for each experimental year, resulting in a balanced selection covering all districts, growth stages, and years included in the study. Single-spore cultures were obtained following Garber et al. (2012). Monosporic isolates were grown in Potato Dextrose Broth (PDB) (for 1 L of PDB, 400 ml of potato-based extract, 20 gr of glucose, sterile water to volume) and incubated at 31 °C on an orbital shaker for two days to promote mycelial growth.

Fungal biomass was collected by centrifugation, and genomic DNA was extracted using the NucleoSpin Plant II kit (Macherey-Nagel, Düren, Germany). DNA yield and purity were assessed with a NanoDrop 2.0 spectrophotometer (ThermoFisher Scientific, Wilmington, DE, USA).

Species determination was achieved by SYBR Green real-time PCR as described by Sardiñas et al. (2011). Each reaction (20 µL final volume) contained 10 µL GoTaq qPCR Master Mix (Promega, Madison, WI, USA, item A6101), 0.6 µL of each species-specific primer pair [stock concentration 10 µM; final concentration 300 nM per primer. *A. flavus*: FLAVIQ1 (5′-GTCGTCCCCTCTCCGG-3′)/FLAQ2 (5′-CTGGAAAAAGATTGATTTGCG-3′); *A. parasiticus*: FLAVIQ1 (5′-GTCGTCCCCTCTCCGG-3′)/PARQ2 (5′-GAAAAAATGGTTGTTTTGCG-3′); Eurofins Scientific, Torino, Italy], 0.4 µL MgCl_2_, 0.16 µL CXR dye, 5 µL template DNA, and nuclease-free water. Amplification was carried out in a StepOnePlus Real-Time PCR System (ThermoFisher Scientific, Wilmington, DE, USA) with the following program: 95 °C for 10 min, followed by 40 cycles of 95 °C for 15 s and 60 °C for 1 min. Melting curve analysis was performed between 60 and 95 °C with 0.3 °C increments every 30 s.

Positive controls (ITEM 8069 - A*. flavus*; ITEM 18299 - A*. parasiticus*), together with negative controls (nuclease-free water) were included in each run. Cross-amplification was tested by running *A. parasiticus* DNA with *A. flavus* primers and *vice versa*. Isolates were assigned to species according to melting temperatures (Tm: 88.5 °C for *A. flavus*, Tm: 89.7 °C for *A. parasiticus*) and curve profiles consistent with those of reference strains.

### 2.6 Aflatoxin analysis

AF detection followed UNI EN ISO 16050:2011 (ISO, 2011) and was performed for BBCH 89 and late-harvest samples only. Briefly, 50 g of hazelnut flour were blended with 10 g sodium chloride, 150 mL methanol, and 100 mL sterile water for 1 min. Extracts were filtered (Falc Instruments, Treviglio, Italy) and diluted 1:1 with sterile water. Ten milliliters of the filtrate were passed through Easi Extract Aflatoxin immunoaffinity columns (R-Biopharm Rhone Ltd, Glasgow, UK) containing monoclonal antibodies against AFB_1_, AFB_2_, AFG_1_, and AFG_2_.

Columns were rinsed twice with 10 mL sterile water, dried with air, and eluted with 1 mL methanol, followed by 1 mL deionized water. Eluates were collected in 2 mL glass vials, adjusted to volume when necessary, and analyzed by reverse-phase HPLC (Vanquish Core, Thermo Scientific, Waltham, MA, USA) with post-column derivatization and fluorescence detection. Separation was performed on a Supelcosil LC-18 column (Merck KGaA, Darmstadt, Germany) with a mobile phase of H_2_O:CH_3_CN: CH_3_OH (3:1:1, *v/v/v*) at 1 mL/min. AF levels were expressed in µg/kg, with a detection limit (LOD) of 0.2 µg/kg for all toxins considered.

### 2.7 Water activity measurements

Water activity (a_w_) was recorded for hazelnuts during the three experimental seasons (2023-2025) using a portable AquaLab Pawkit meter (Graintec Scientific, Toowoomba, QLD, Australia). Measurements were taken weekly starting from the onset of the BBCH 79 stage until the end of BBCH 89 stage, to monitor kernel a_w_ dynamic under field conditions. For each geographical area, triplicate determinations were carried out on reference hazelnut samples collected from selected orchards representative of the same production areas as the monitored orchards, to capture local field conditions. In addition, a_w_ was measured immediately after collection for each of the six late-harvest hazelnut samples, to assess kernel status at harvest.

### 2.8 Statistical analysis

Statistical analyses were performed with IBM SPSS Statistics software, version 30.0 (Chicago, IL, USA). Data on *A.* section *Flavi*, *Alternaria* spp., *A.* section *Nigri*, *Fusarium* spp. and *Penicillium* spp. CFU/g of inflorescence on hazelnut kernel were Log_10_ transformed before statistical analysis to homogenize the variance. CFU/g for each fungus were subjected to ANOVA. Factors included were “geographical area (G)”, “year (Y)” and “growth stage (GS)”, with their interactions. Tukey’s test was used to compare means and identify significant differences (*P ≤ 0.05*). Heatmaps were generated using the R statistical environment with the *pheatmap* package, based on Log_10_(CFU + 1) transformed data, exclusively for graphical visualization.

## 3 Results

### 3.1 Environmental variables

Meteorological conditions recorded across the three Azerbaijani production areas during 2023–2025 are summarized in **Table 1**. The general climatic pattern was consistent with the humid subtropical-continental gradient typical of the districts, with mild winters and warm to hot summers.

**Table 1:** Summary of meteorological data collected during 4 periods from the 1st of January to 30th August (P1: 1 Jan - 30 Apr; P2: 1 May - 30 June; P3: 1 July - 31 July; P4: 1 Aug - 31 Aug) in different regions of Azerbaijan (Khachmaz, Qabala, Zaqatala) in years 2023-2025.

Geographical area	Phase	Mean air temperature (Tm, °C)^1^	Degree day (DD, °C)^2^	Summation of degree day (ΣDD, °C)^3^	Total rainfall (R, mm)^4^	Summation of rainfall (ΣR, mm)^5^	Mean air relative humidity (RHm, %)^6^
2023							
Khachmaz							
	P1	6.5	780	780	1.8	1.8	84
	P2	20.0	1221	2001	1.2	3.1	76
	P3	25.4	787	2788	0.5	3.6	67
	P4	26.5	822	3610	0.2	3.7	59
Qabala							
	P1	7.4	883	883	5.5	5.5	71
	P2	20.6	1258	2141	9.5	15.0	67
	P3	25.4	787	2929	3.6	18.6	60
	P4	26.7	829	3758	1.0	19.6	53
Zaqatala							
	P1	8.3	998	998	8.7	8.7	65
	P2	20.7	1260	2257	8.5	17.2	66
	P3	25.4	787	3044	3.8	21.0	58
	P4	26.8	830	3874	2.8	23.8	57
2024							
Khachmaz							
	P1	7.0	841	841	2.3	2.3	79
	P2	20.2	1229	2071	2.1	4.4	71
	P3	25.0	775	2846	1.2	5.6	71
	P4	24.6	762	3608	0.3	5.9	68
Qabala							
	P1	7.4	901	901	8.6	8.6	73
	P2	20.2	1230	2131	14.7	23.3	67
	P3	25.5	790	2921	5.4	28.7	55
	P4	25.8	798	3719	1.3	30.0	53
Zaqatala							
	P1	9.2	1112	1112	9.3	9.3	68
	P2	20.6	1255	2367	16.4	25.6	70
	P3	25.4	786	3153	5.4	31.0	63
	P4	25.5	791	3944	2.5	33.5	63
2025							
Khachmaz							
	P1	6.2	739	739	6.2	6.2	83
	P2	19.3	1180	1919	0.8	7.0	76
	P3	25.9	803	2722	0.1	7.0	64
	P4	25.4	786	3508	0.4	7.5	82
Qabala							
	P1	6.8	818	818	9.3	9.3	73
	P2	20.3	1238	2056	3.1	12.4	62
	P3	27.3	846	2901	0.7	13.1	45
	P4	27.0	836	3738	0.4	13.6	46
Zaqatala							
	P1	7.8	935	935	10.8	10.8	71
	P2	20.3	1237	2172	5.9	16.7	68
	P3	26.6	826	2997	1.9	18.6	58
	P4	26.7	826	3824	0.1	18.7	57

^1^Mean air temperature (Tm, °C) was calculated as the mean of the temperatures for each period;.

^2^Degree Days (DD, °C) was computed as the sum of the mean daily T;

^3^Summation of DD (ΣDD, °C) was obtained by adding the DD of each period;

^4^Total R (R, mm) was computed as the sum of daily R;

^5^Summation of R (ΣR, mm) was obtained by adding R of each period;

^6^Mean RH (RHm, %) was calculated as the mean of the RH for each period.

In P1, Tm ranged from 6.2 °C in Khachmaz (2025) to 9.2 °C in Zaqatala (2024), with ΣDD between 739 and 1112. This period was generally wetter in the western areas (Qabala and Zaqatala), where R exceeded 8 mm in most years, compared to <3 mm in Khachmaz. RHm was also higher in the coastal Khachmaz area (79-84%) than inland (65-73%).

In P2, Tm increased to around 19-21 °C across all locations, with ΣDD values between 1919 and 2367. R was again greater in Qabala and Zaqatala (up to 16 mm in 2024) compared to Khachmaz (<3 mm), reflecting stronger continental precipitation patterns toward the mountains.

P3 and P4 periods were characterized by peak Tm (25-27 °C) and ΣDD between 2700 and 3900. R was very limited during these months, with totals below 6 mm in all sites, confirming the common occurrence of dry summer in the area. RHm progressively decreased through the season, reaching its lowest values in Qabala (45-46%) and Zaqatala (57-58%) during August, while remaining slightly higher in Khachmaz (64-68%).

Overall, interannual variability was moderate, with 2024 standing out for higher temperatures and precipitation totals, especially in Qabala and Zaqatala, while 2025 showed reduced cumulative rainfall, most notably in phases P3 and P4. The ΣDD from January to August ranged between 3500 and 3900 °C across all sites, indicating broadly similar thermal regimes.

The differences in climatic conditions, together with cultivar differences, reflected in the hazelnuts sampling dates, that varied slightly among districts. In Khachmaz, each growth stage was generally reached about two weeks earlier than in Qabala and Zaqatala. Specifically, BBCH 70 was sampled in early April in Khachmaz and in late April in the other two districts; BBCH 71 in early May *versus* late May; BBCH 79 in early June *versus* late June; and BBCH 89 between early-mid August in Khachmaz and late August in Qabala and Zaqatala.

ANOVA supported the climatic patterns described above, showing significant differences among time periods for Tm and ΣDD (*P* < 0.01) and among periods and geographical areas for RHm (*P* < 0.01), while no significant differences among years were detected (*P* > 0.05) (**Supplementary Table 2**).

### 3.2 Pre-harvest fungal occurrence

ANOVA results showed that fungal occurrence in hazelnuts, before harvest, was significantly affected by geographical area, year, and growth stage, as well as by their interactions (**Table 2**).

**Table 2:** Univariate analysis of variance (ANOVA) of Log_10_(CFU/g) values for *Aspergillus* section *Flavi*, *Aspergillus* section *Nigri*, *Alternaria* spp., *Fusarium* spp., and *Penicillium* spp., obtained from hazelnut and inflorescence sample collected in thirty orchards in Azerbaijan.

Log_10_(CFU/g)										
Factors	*A.* section *Flavi*		*A.* section *Nigri*		*Alternaria* spp.		*Fusarium* spp.		*Penicillium* spp.	
Geographical area (G)	**		**		n.s		n.s		n.s	
Khachmaz	1.0	_b_	1.4	_b_	2.1		2.0		2.3	
Qabala	1.6	_a_	1.8	_a_	1.9		2.3		2.3	
Zaqatala	1.7	_a_	1.5	_ab_	1.8		2.1		2.4	
Year (Y)	**		**		**		**		**	
2023	1.0	_b_	1.3	_b_	1.6	_b_	1.4	_b_	2	_b_
2024	1.6	_a_	1.6	_a_	2.2	_a_	2.4	_a_	2.1	_b_
2025	1.8	_a_	1.6	_a_	1.9	_a_	2.4	_a_	3	_a_
Growth Stage (GS)	**		**		**		**		**	
BBCH 70	1.2	_b_	1.1	_bc_	2.9	_a_	1.6	_b_	1.6	_b_
BBCH 71	1.0	_b_	1	_c_	2.8	_a_	2.2	_a_	3.1	_a_
BBCH 79	2.4	_a_	2.5	_a_	0.5	_c_	2.2	_a_	2.9	_a_
BBCH 89	1.2	_b_	1.4	_b_	1.4	_b_	2.4	_a_	1.9	_b_
Interactions										
G x Y	**		**		n.s		**		n.s	
G x GS	**		**		*		**		n.s	
Y x GS	**		**		n.s		**		**	
G x Y x GS	**		**		n.s		**		**	

**P < 0.01; *P < 0.05; n.s for not significant. Different letters indicate significant differences according to Tukey’s test.

Samples were sampled in four different growth stages (BBCH 70, BBCH71, BBCH 79 and BBCH 89), across three consecutive cropping seasons (2023, 2024, 2025), in three regions of Azerbaijan (Khachmaz, Qabala and Zaqatala).

**Figure 2:**
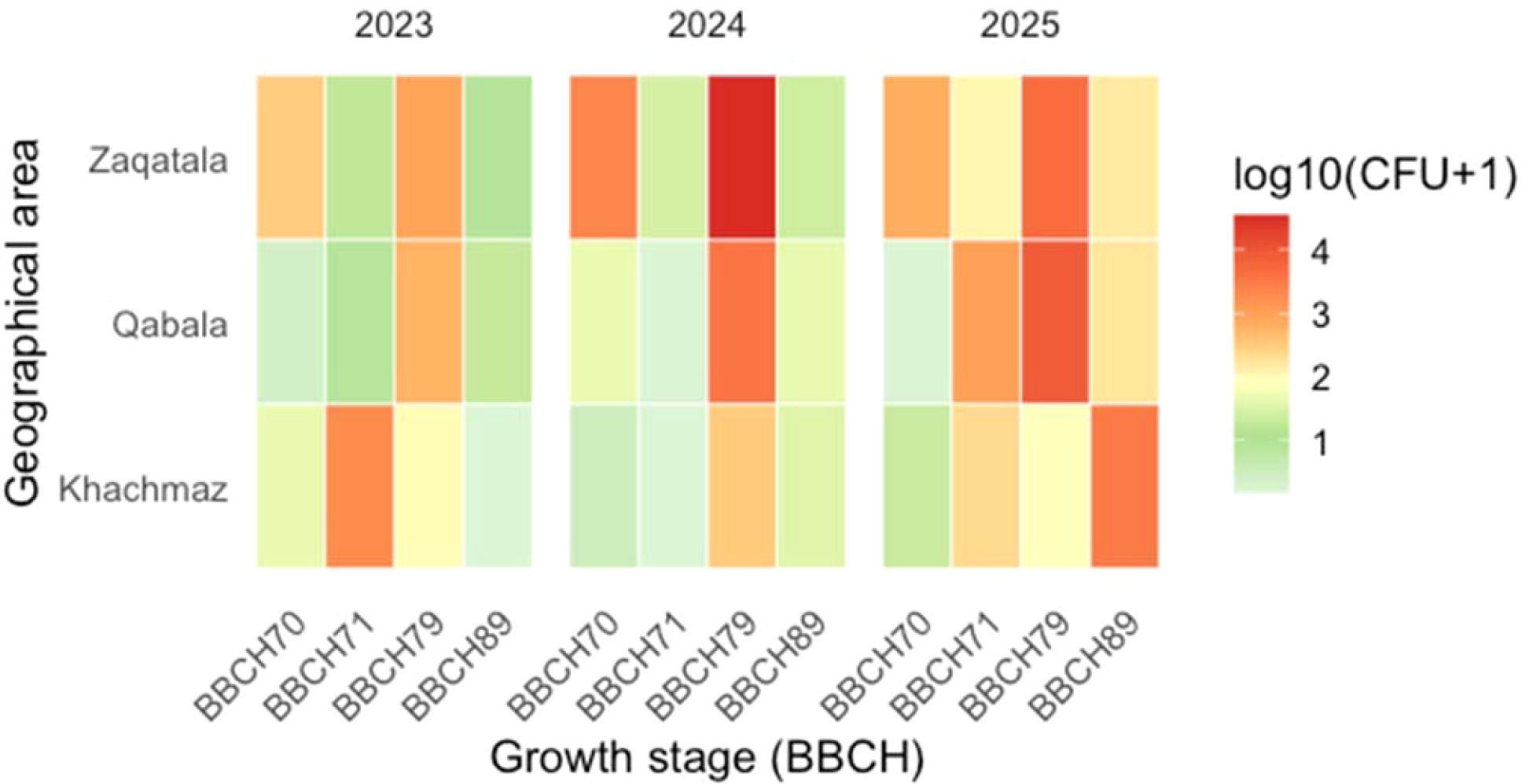
Distribution of fungal contamination in hazelnut samples at four developmental stages (BBCH 70–89) in Khachmaz, Qabala, and Zaqatala. Fungal groups are displayed on the y-axis, and BBCH stages on the x-axis. Heatmap colors reflect fungal load (Log_10_(CFU + 1)), emphasizing shifts in dominance among *Aspergillus* spp., *Alternaria* spp., *Fusarium* spp., and *Penicillium* spp. during nut development.

**Figure 3:**
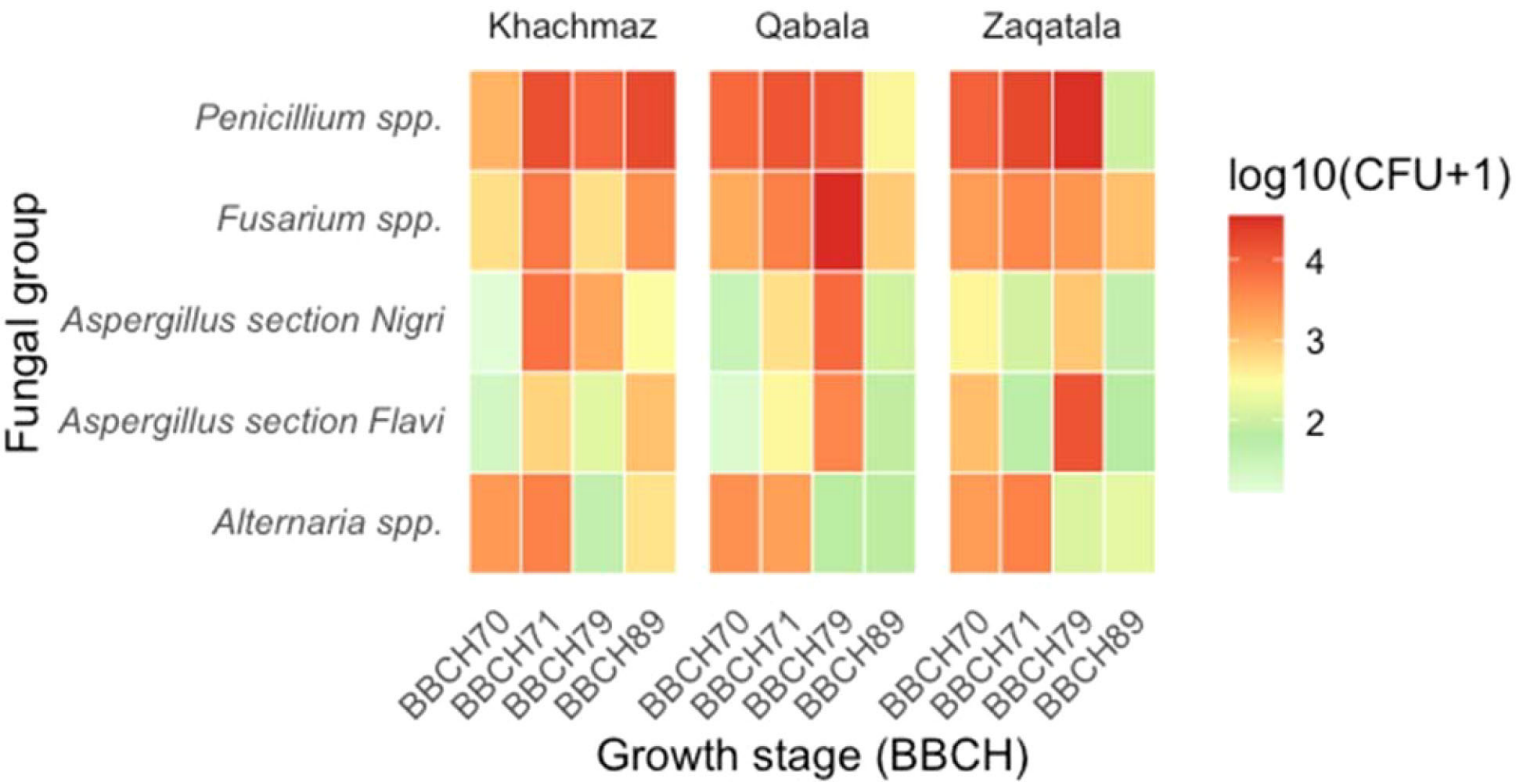
Heatmap illustrating the interaction between geographical area (Khachmaz, Qabala, Zaqatala), growth stage (BBCH 70, 71, 79, 89) and sampling year (2023–2025) on the abundance of *Aspergillus* section *Flavi* in hazelnut samples. Each facet represents one sampling year, with rows indicating the three production areas and columns the four phenological stages. Color intensity reflects fungal load expressed as Log_10_(CFU + 1), following a soft green-to-red gradient to indicate increasing infestation levels.

For *A.* section *Flavi*, geographical location played a central role (*P* < 0.01), with higher loads in Qabala (1.6 Log_10_ CFU/g) and Zaqatala (1.7 Log_10_ CFU/g) compared to Khachmaz (1.0 Log_10_ CFU/g). Temporal dynamics revealed a progressive increase in occurrence levels, from relatively low values in 2023 (1.0 Log_10_ CFU/g) to markedly higher loads in 2025 (1.8 Log_10_ CFU/g). Growth stage also had a pronounced effect: fungal occurrence peaked at the immature fruit stage (BBCH 79, 2.4 Log_10_ CFU/g), while both early (BBCH 70-71) and final (BBCH 89) stages showed lower values. Importantly, several interactions were significant (G x Y, G x GS, Y x GS, and G x Y x GS), highlighting that geographical effects were not uniform across years or growth stages. These patterns suggest that the influence of location reflects the combined effects of environmental conditions and fruit growth stage, a relationship qualitatively illustrated by the heatmap plots (**Figures 2**, **3**).

For *A.* section *Nigri*, patterns were largely comparable. Occurrence was significantly higher in Qabala (1.8 Log_10_ CFU/g), increased sharply from 2023 to 2024-2025 (1.6 Log_10_ CFU/g), and reached its maximum at BBCH 79 (2.5 Log_10_ CFU/g). This confirms that both *A.* section *Flavi* and *A.* section *Nigri* thrive under similar environmental and crop phenology conditions.

Other fungal genera showed more specific dynamics. *Alternaria* spp. exhibited an opposite trend, being most abundant at early stages (2.9 Log_10_ CFU/g) and decreasing sharply with fruit maturation. *Fusarium* spp. and *Penicillium* spp. displayed less consistent patterns, but they were mainly influenced by temporal and phenological factors: both fungal genera increased significantly between 2023 and 2025, with *Fusarium* spp. peaking at later stages (BBCH 89, 2.4 Log_10_ CFU/g) and *Penicillium* spp. showing the highest loads at BBCH 71 (3.1 Log_10_ CFU/g).

When considering the relative incidence of fungal genera, distinct patterns emerged among geographic areas, years, and developmental stages (**Table 3**).

**Table 3:** Relative incidence (%) of *Aspergillus* sections *Flavi* and *Nigri*, *Alternaria* spp., *Fusarium* spp., *Penicillium* spp., and other fungi isolated from pre-harvest hazelnut samples, grouped by geographical area (Khachmaz, Qabala, Zaqatala), year (2023, 2024, 2025), and fruit growth stage (BBCH 70, 71, 79, 89).

Relative incidence (%)							
Factors	*A.* section *Flavi*	*A.* section *Nigri*	*Alternaria* spp.	*Fusarium* spp.	*Penicillium* spp.	Others	Total
Geographical area (G)							
Khachmaz	2.6	10.2	11.3	13.2	58.7	4.0	100
Qabala	4.5	5.4	8.2	42.0	35.3	4.6	100
Zaqatala	13.8	6.8	1.4	9.5	57.3	11.3	100
Year (Y)							
2023	1.2	10.8	2.2	11.5	53.9	20.4	100
2024	40.8	10.9	6.1	19.1	20.4	2.8	100
2025	4.4	2.7	6.6	20.9	65.1	0.4	100
Growth Stage (GS)							
BBCH 70	4.5	19.9	1.5	13.2	53.8	7.1	100
BBCH 71	0.9	12.6	5.8	14.1	52.6	14.1	100
BBCH 79	18.5	0.2	5.7	19.9	52.4	3.3	100
BBCH 89	4.0	3.0	1.5	19.3	56.7	15.6	100

“Others” includes fungal taxa detected during isolation that did not belong to the targeted mycotoxigenic genera. These isolates were not further identified or characterized, as they were outside the scope of the present study. Based on colony morphology, they mainly included non-mycotoxigenic genera such as *Rhizopus* and *Trichoderma* spp., and sporadically other *Aspergillus* species (e.g. *A. ochraceus*).

Across locations, *Penicillium* spp. dominated the overall mycobiota, representing more than half of the total isolates in Khachmaz (58.7%) and Zaqatala (57.3%), and slightly less in Qabala (35.3%). *Fusarium* spp. were also abundant, particularly in Qabala (42.0%), where they co-occurred with a notable proportion of *A. section Nigri* (8.2%) and *A.* section *Flavi* (4.5%). In contrast, Khachmaz was characterized by the highest relative abundance of *A.* section *Nigri* (11.3%) and *Penicillium*, while *A.* section *Flavi* remained minor (2.6%). Interestingly, Zaqatala showed the highest incidence of *A.* section *Flavi* (13.8%).

Temporal trends further supported these observations. The composition of the fungal community varied markedly between years, with *A.* section *Flavi* being relatively scarce in 2023 (1.2%) and 2025 (4.4%) but dominating in 2024 (40.8%). In contrast, *Penicillium* spp. were consistently prevalent across all years, while *Alternaria* spp. and *Fusarium* spp. showed intermediate and variable occurrence.

Across growth stages, *Penicillium* spp. remained dominant (50–57%), whereas *A.* section *Flavi* displayed a marked increase at the ripening stage (BBCH 79; 18.5%). Conversely, *Alternaria* spp. prevailed at the earliest stages (up to 19.9% at BBCH 70–71) and declined thereafter.

Considering the late-harvest lots, they revealed a marked increase in *Aspergillus* occurrence, particularly within section *Flavi*, in both on-tree and ground-collected nuts. Log_10_(CFU/g) counts ranged between 3.5 and 4.7, representing a substantial rise compared to the regular harvest of the same season. Fungal occurrence was consistently higher in ground-collected nuts. *A.* section *Nigri* followed a similar pattern, though at lower magnitudes, while *Fusarium* spp. showed sporadic but pronounced peaks (up to 5.2 Log_10_(CFU/g)). Conversely, *Alternaria* spp. was almost absent (**Supplementary Table 3**).

### 3.3 Molecular confirmation of *A. flavus* and *A. parasiticus* isolates

Isolates classified within *Aspergillus* section *Flavi* exhibited uniform morphological traits when cultured on 5/2 agar. Most colonies developed the typical ivy-green pigmentation, with a granular to powdery texture and pale yellow to orange coloration on the reverse, features consistent with *A. flavus*. In contrast, a single isolate showed a cress-green, velvety surface, characteristic of *A. parasiticus* (Rapper and Fennel, 1965).

Stereomicroscopic examination revealed that *A. flavus*-like colonies had densely sporulating surfaces and frequently produced sclerotia, whereas the *A. parasiticus*-like isolate lacked these structures. Wet-mount preparations confirmed the presence of unbranched conidiophores with globose vesicles and rough-walled conidia, confirming the morphological traits typical of the genus *Aspergillus*.

Subsequent molecular analysis by SYBR Green real-time PCR supported these observations confirming all isolates as *A. flavus*, except for one *A. parasiticus*, isolated originally in 2023 from hazelnuts cultivated in Khachmaz area. In one case, a single *Aspergillus* isolate not belonging to *A. flavus* or *A. parasiticus* was detected; however, due to its sporadic occurrence, it was not further characterized.

### 3.4 Aflatoxin contamination

Across the three monitored production years (2023-2025), AF concentrations in pre-harvest hazelnut samples were generally low, with most lots showing values below the European regulatory limits. Contamination levels remained limited in 2023 and below LOD in 2024, while a wider variability was observed in 2025, when detectable amounts of both B- and G-type AF were found in two samples from Khachmaz district. No contamination was detected in samples collected from Qabala and Zaqatala during the same period (**Table 4**).

**Table 4:** Aflatoxin concentrations (AFB_1_, AFB_2_, AFG_1_, AFG_2_, and total aflatoxins) in hazelnut samples collected at BBCH 89 and late-harvest periods across different years and locations in Azerbaijan.

Sample code	Collection date	Year	Location	Collection point	AFB1 (μg/kg)	AFB2 (μg/kg)	AFG1 (μg/kg)	AFG2 (μg/kg)	AF tot (μg/kg)	AF status (EU limit)
K29 n6	08-Aug	2023	Khachmaz	Tree	1.2	2.2	0.7	1.3	5.4	Compliant
K2 n4	08-Aug	2025	Khachmaz	Tree	123.3	19.9	95.7	12.4	251.3	Non-compliant
K3 n5	08-Aug	2025	Khachmaz	Tree	0.5	0.2	0.5	0.2	1.4	Compliant
ZRC n39	06-Sept	2025	Zaqatala	Ground	509.1	54.1	167.3	22.3	752.8	Non-compliant
ZRC n39	06-Sept	2025	Zaqatala	Tree	183.9	11.1	83.5	7.1	285.6	Non-compliant
Zaqatala 1	10-Sept	2025	Zaqatala	Ground	183.4	12.5	47.8	5.9	249.6	Non-compliant
Zaqatala 1	10-Sept	2025	Zaqatala	Tree	320.1	33.7	268.6	27.2	649.6	Non-compliant
Zaqatala 2	11-Sept	2025	Zaqatala	Ground	40.1	6.5	18.7	5.9	71.2	Non-compliant
Zaqatala 2	11-Sept	2025	Zaqatala	Tree	94.9	5.6	31.5	3.3	135.3	Non-compliant

AF status was assigned according to Commission Regulation (EU) No 2023/915, using the maximum limits established for hazelnuts intended for direct human consumption (5 µg/kg for AFB1 and 10 µg/kg for total aflatoxins).

Late-harvest samples (September 2025) include paired collections from tree and ground nuts to assess the impact of delayed harvest on aflatoxin contamination. Aflatoxin concentrations are expressed in µg/kg.

In 2025, the additional set of six late-harvest samples, collected to assess the effect of delayed harvesting, presented AF levels markedly higher than in regular harvest samples. AFB_1_ ranged between 40.1 µg/kg and 509.1 µg/kg, and total AF reached up to 752.8 µg/kg in ground-collected nuts. Samples collected from trees showed elevated levels too, with AFB_1_ between 94.9 µg/kg and 320.1 µg/kg. Also in this case, both B- and G-type AF were detected across most late-harvest samples.

### 3.5 Water activity

Water activity values were monitored weekly from the beginning of fruit filling (BBCH 79) to full maturity (BBCH 89) (**Table 5**). In 2023, a_w_ remained close to saturation throughout nut development, with mean values of 0.99 in Gebele and Zaqatala and 0.98 in Khachmaz, decreasing at harvest (0.92-0.95). The 2024 season showed lower overall a_w_, especially in Zaqatala, where values declined from 0.95 during BBCH 79 to 0.93 at harvest and 0.77 at the final observation in early September. In 2025, Khachmaz maintained nearly constant a_w_ (≈ 0.98) throughout the season, while Zaqatala again exhibited a progressive decrease from 0.91 to 0.88.

**Table 5:** Water activity (a_w_) of hazelnut kernels during BBCH 79, BBCH 89, and at the end of the monitoring period (final measurement) in the three surveyed regions (Khachmaz, Qabala, Zaqatala), from 2023–2025.

Year	Location	BBCH 79	BBCH 89	Final a_w_
2023	Khachmaz	0.98	0.98	0.92
	Qabala	0.99	0.99	0.95
	Zaqatala	0.99	0.99	0.94
2024	Khachmaz	0.98	0.96	0.96
	Zaqatala	0.95	0.93	0.77
2025	Khachmaz	0.98	0.98	0.97
	Zaqatala	0.91	0.83	0.79

For year 2024 and 2025, a_w_ measures for Qabala are not available.

The measures correspond to the mean a_w_ value registered in the period in which the BBCH was observed in the orchard.

Additional late-harvest samples collected in September 2025 confirmed this seasonal decline in kernel a_w_. For the ZRC n39 orchard, a_w_ measured 0.92 in tree-collected nuts and 0.87 in ground-collected nuts. In the first Zaqatala orchard, values were a_w_ = 0.87 (tree) and a_w_ = 0.82 (ground), whereas in the second one they were a_w_ = 0.87 (tree) and a_w_ = 0.93 (ground).

## 4 Discussion

Results of this research showed how a relevant fungal occurrence in hazelnuts before harvest, strongly influenced by geographical area and growth stage, and modulated by interannual climatic variability, is widespread across production systems, with *A*. section *Flavi* and *Nigri* showing the most pronounced and consistent responses to environmental variation. However, aflatoxin contamination risk was not directly associated with fungal occurrence alone but emerged only under specific environmental and phenological conditions.

Across the three-year survey, the presence of *A.* section *Flavi* increased progressively from 2023 to 2025, particularly in the Qabala and Zaqatala districts, which consistently exhibited higher fungal loads and prevalence compared to Khachmaz. Relative abundance data confirmed this spatial pattern: *A.* section *Flavi* represented up to 14% of the total isolates in Zaqatala, against less than 5% in the other areas. These differences can be attributed to district climatic characteristics. Both Qabala and Zaqatala showed higher ΣDD and lower RHm during nut ripening, particularly in late summer (P3–P4). Warm, dry conditions are widely recognized as favorable to *A. flavus* growth, and the observed trends support a strong climatic influence on fungal proliferation and the establishment of conditions conducive to toxin synthesis (Park and Bullerman, 1983; Schroeder and Hugo Hein, 1968; Trucksess et al., 1988).

Temporal patterns reinforced this relationship. Differences in fungal occurrence observed across years corresponded with warmer and drier conditions during late summer, particularly in the P3–P4 periods. While 2024 showed intermediate conditions, the 2025 season was characterized by markedly a warmer late summer, especially in Qabala and Zaqatala, where Tm exceeded 26 °C and RH was below 60%. These conditions coincided with the highest *A.* section *Flavi* counts and AF levels, highlighting the role of climatic stress in creating favorable conditions for aflatoxin biosynthesis rather than indicating a direct quantitative relationship between fungal abundance and toxin concentration.

Phenological patterns further clarified the infection dynamics. Infection by *A.* section *Flavi* was detectable since BBCH 70 and peaked at the immature fruit stage (BBCH 79). This trend indicates how infection likely starts during the flowering period and progresses during the nut-filling phase, when shells are hardened but the kernel retains sufficient moisture to sustain fungal development. This agrees with previous observations indicating that colonization by *Aspergillus* spp. may occur during the reproductive period but becomes particularly critical immediately preceding harvest (Ozay et al., 2008). A similar pattern was observed for *A.* section *Nigri*, suggesting overlapping ecological niches between these xerophilic fungi. In contrast, *Alternaria* spp. were predominant in the early stages (BBCH 70–71), consistent with their opportunistic behavior on young, green tissues that are later colonized by more stress-tolerant genera such as *Aspergillus* and *Penicillium* spp.

The fungal community observed in this study was broadly consistent with previous surveys conducted on hazelnuts in major producing countries. As reported by Şen and Kabak (2025), hazelnut kernels typically host a diverse mycobiota dominated by *Penicillium*, *Aspergillus*, *Fusarium*, and *Alternaria* spp., together with occasional isolates of *Rhizopus*, *Cladosporium*, *Diaporthe* and *Geotrichum* spp (Arciuolo et al., 2020; Habibi and Afzali, 2021; Şimşek et al., 2002). Similarly, a multi-country survey of ready-to-eat hazelnuts from Turkey, Italy, Spain, and the United States found *Penicillium* (38%) and *Aspergillus* (19%) as the most frequently detected genera (Lombardi et al., 2022). Results of this research reflected this general pattern: *Penicillium* spp. dominated across all sampling sites, whereas *A.* section *Flavi* accounted for a smaller yet consistent fraction of the total fungal population, particularly in the warmer districts of Qabala and Zaqatala. In addition, molecular analyses provided insights into the species composition within *A*. section *Flavi*, confirming the largely dominant presence of *A. flavus* but also the occurrence of *A. parasiticus*, a finding consistent with the detection of both B- and G-type aflatoxins in contaminated samples.

These findings indicate that the high prevalence of *A.* section *Flavi* in the field does not necessarily translate into aflatoxin contamination. Instead, toxin production appears to be primarily regulated by kernel physiological status and environmental stress conditions occurring during the final stages of ripening. Quantification of AF confirmed that, under typical pre-harvest conditions, contamination risk in hazelnut orchards remains minimal. In samples collected at the proper maturity stage (BBCH 89), AFB_1_ concentrations were consistently below the maximum limit set by the European Commission for unprocessed hazelnuts, reflecting that adequate orchard management and timely harvest effectively limited AF biosynthesis. The absence of relevant toxin amounts, despite the frequent isolation of *A.* section *Flavi* in field samples, can be largely explained by the high a_w_ values recorded during nut maturation, which remained close to 0.99 until late August and rarely dropped below 0.90 before harvest. Such high kernel a_w_ supports fungal colonization but does not favor AF production, as toxin synthesis by *A. flavus* is generally limited at a_w_ > 0.95 (Giorni et al., 2008). This observation was consistent with the findings of Ozay et al. (2008), who reported that *A. flavus* and *A. parasiticus* were commonly detected in Turkish hazelnuts during nut development, yet AF contamination in field samples remained very low, generally below 1 µg/kg at harvest. In both studies, contamination increased only when nuts remained in contact with the soil or when post-harvest drying was delayed, emphasizing the critical role of kernel drying in limiting toxin accumulation (Casu et al., 2026).

In contrast, the six late-harvest samples collected in 2025 revealed a markedly different scenario. Nuts harvested approximately three weeks after physiological maturity exhibited substantially higher AF concentrations, with AFB_1_ frequently above 200 µg/kg, with 1 sample exceeding 500 µg/kg and total AF surpassing 700 µg/kg. These samples, originating from the Zaqatala area, were characterized by mean kernel a_w_ values between 0.82 and 0.93, combined with air Tm above 26 °C and RHm below 60%. Such conditions fall within the optimal range for AF biosynthesis, highlighting how even a moderate reduction in kernel a_w_, when coupled with heat stress during the late pre-harvest phase, can trigger toxin production.

Ground contact has been widely identified as a major driver of AF contamination in hazelnuts, as prolonged contact with moist soil favors fungal growth and toxin production (Ozay et al., 2008; Pscheidt et al., 2019). However, an important observation from this study is that elevated AF levels were also detected in late-harvest nuts collected directly from the tree, in some cases higher than those measured in ground-collected samples. This confirms that, under favorable climatic conditions combined with late harvest, AF biosynthesis may already be initiated before nuts reach the soil, during the final stages of ripening.

The number of late-harvest samples analyzed was limited, and therefore these results do not allow for definitive conclusions. Nevertheless, they indicate that delayed harvest alone, irrespective of soil contact, may represent a critical risk factor when combined with heat stress and reduced kernel a_w_, important message for hazelnut value chain stakeholders.

Similarly, Şen and Kabak (2025) emphasized that hazelnuts’ thick shells act as a protective barrier that limits fungal penetration during early fruiting, and that AF contamination arises mainly under combined conditions of crop water stress, insect damage, and elevated temperature. Although this study did not include an evaluation of insects activity, previous research in hazelnuts and other nut crops, such as pistachios and almonds, has demonstrated the key role of insects not only as vectors of *A. flavus* spores but also as agents causing kernel damage and wounds that facilitate fungal penetration and colonization, thereby increasing the risk of AF contamination (Doster and Michailides, 1994a; Fontana et al., 2014; Palumbo et al., 2014; Picot et al., 2016; Thomson and Mehdy, 1978).

It is important to note that this study did not investigate agronomic interventions or orchard-management strategies; therefore, no direct conclusions can be drawn regarding the effectiveness of specific GAPs. The only management-related factor addressed experimentally was harvest timing, and late-harvest samples clearly enhanced markedly increases of AF contamination risk. However, broader GAP principles, including adequate fertilization, canopy pruning, timely removing of decaying plant debris and irrigation management, remain central elements of AF prevention (Marín and Ramos, 2016; Şen and Kabak, 2025).

Beyond GAPs, biological control represents one of the most effective pre-harvest tools to manage AF risk. The application of biocontrol formulations based on atoxigenic strains of *A. flavus* has been successfully used in several crops, including perennial tree crops such as pistachio and almond, where these strains competitively exclude toxigenic populations and substantially reduce AF accumulation (Cotty, 2006; Dorner, 2004; Doster et al., 2014). Although no such products are currently registered for hazelnut, these research findings highlight the value of biocontrol strategies for hazelnut orchards, particularly in areas such as Azerbaijan where environmental conditions favor *A.* section *Flavi* activity. In recent years, preliminary surveys in Azerbaijan have already highlighted the presence of naturally occurring atoxigenic *A. flavus* genotypes, suggesting that biocontrol strategies could be adapted to local orchard ecosystems (Aghayev et al., 2025).

Altogether, these results support a conceptual framework in which pre-harvest AF contamination in hazelnuts arises from the interplay between widespread fungal colonization, environmental stress conditions, and the critical phase surrounding harvest. While *A.* section *Flavi* is commonly present in orchards, aflatoxin biosynthesis is triggered only when late-season heat and reduced kernel water activity coincide with delayed harvest. These findings underscore the importance of timely harvest and careful handling during the final ripening stages as key leverage points for minimizing risk. In this framework, complementary preventive strategies, such as biological control and the development of predictive tools tailored to hazelnut systems, may further enhance AF management under present and future climatic scenarios.

## Data Availability

The original contributions presented in the study are included in the article/**Supplementary Material**. Further inquiries can be directed to the corresponding author/s.
