# Supplementary material for: Pre-harvest dynamics of Aspergillus section Flavi and aflatoxin risk in hazelnut orchards of Azerbaijan
Source: Front Plant Sci. 2026 Mar 5;17:1791562. doi: 10.3389/fpls.2026.1791562 (PMC13001648; doi:10.3389/fpls.2026.1791562)
Supplement: Supplementary file 1 [file Table1.docx]

Supplementary Material

**Supplementary Table S1.** Hazelnut orchards surveyed during the three-year study period (2023-2025). Each code corresponds to a distinct orchard located in Azerbaijan (Khachmaz, Zaqatala, and Qabala regions). For each orchard, the table reports the region, village, village latitude and longitude, and orchard size in hectares.

| **Orchard Code** | **Region** | **Village** | **Latitude** | **Longitude** | **Ha** | **Dominant**  **Cultivar** |
| --- | --- | --- | --- | --- | --- | --- |
| K33 n2 | Khachmaz | Xaspoladoba | 41.2924 | 48.4938 | 6.5 | Khachmaz |
| K2 n4 | Khachmaz | Armudpadar | 41.2627 | 48.4605 | 2 | Khachmaz |
| K3 n5 | Khachmaz | Yergüc | 41.2529 | 48.4423 | 1.35 | Khachmaz |
| K29 n6 | Khachmaz | Çinartala | 41.3255 | 48.4300 | 1 | Khachmaz |
| K31 n7 | Khachmaz | Dədəli | 41.3425 | 48.4546 | 2.52 | Khachmaz |
| K32 n9 | Khachmaz | İdrisoba | 41.3740 | 48.3711 | 2 | Khachmaz |
| Kagr n10 | Khachmaz | Maqsudkənd | 41.3928 | 48.3650 | / | Khachmaz |
| Kagr n12 | Khachmaz | Maqsudkənd | 41.3928 | 48.3650 | / | Khachmaz |
| G23 n16 | Qabala | Kötüklü | 40.5921 | 47.4158 | 0.54 | Ata-baba |
| G19 n17 | Qabala | Sincan | 40.5655 | 47.3435 | 0.5 | Ata-baba |
| G21 n18 | Qabala | Xaçmaz | 41.0259 | 47.3540 | 0.8 | Ata-baba |
| G22 n19 | Qabala | Xaçmazqışlaq | 41.0129 | 47.3622 | 1.2 | Ata-baba |
| G20 n20 | Qabala | Şirvanlı | 41.0103 | 47.2955 | 0.6 | Ata-baba |
| GRC n43 | Qabala | Zarağan | 40.5612 | 47.4856 | / | Ata-baba |
| Zq18 n21 | Zaqatala | Cəlayir | 41.1914 | 46.4751 | 0.33 | Ata-baba |
| Zq44 n23 | Zaqatala | Cəlayir | 41.1914 | 46.4751 | 0.5 | Ata-baba |
| Zq17 n24 | Zaqatala | Tasmalı | 41.2526 | 46.4208 | 2.35 | Ata-baba |
| Z27 n26 | Zaqatala | Əliabad | 41.2832 | 46.3721 | 1 | Ata-baba |
| Zb13 n28 | Zaqatala | Kortala | 41.3706 | 46.2326 | 1 | Ata-baba |
| Zb35 n29 | Zaqatala | İtitala | 41.3445 | 46.2204 | 2 | Ata-baba |
| Zb8 n30 | Zaqatala | Püştətala | 41.3830 | 46.2545 | 2.65 | Ata-baba |
| Zb10 n31 | Zaqatala | Hənifə | 41.4255 | 46.2341 | 0.6 | Ata-baba |
| Z1 n33 | Zaqatala | Fındıqlı | 41.3501 | 46.2700 | 2 | Ata-baba |
| Z6 n35 | Zaqatala | Aşağı Tala | 41.3628 | 46.3809 | 0.8 | Ata-baba |
| Z14 n36 | Zaqatala | Maqov | 41.3545 | 46.2948 | 0.6 | Ata-baba |
| Z7 n37 | Zaqatala | Danaçı | 41.3312 | 46.2554 | 1.2 | Ata-baba |
| Z41 n38 | Zaqatala | Zəyəm | 41.2443 | 46.4053 | 2.5 | Ata-baba |
| ZRC n39 | Zaqatala | Aşağı Tala | 41.3628 | 46.3809 | / | Ata-baba |
| Zagr n40 | Zaqatala | Dağlı | 41.3132 | 46.3137 | / | Ata-baba |
| Zagr n42 | Zaqatala | Dağlı | 41.3132 | 46.3137 | / | Ata-baba |

Notes:

Cultivar information is reported based on dominant cultivars identified at the area level. All orchards included in the study were managed under low-input conditions, as reported by local producers. Due to the heterogeneous and partially undocumented agronomic practices, field-level classification of phytosanitary management was not possible. Management practices are therefore described qualitatively in the manuscript and were not used as stratifying variables in the analyses. “/” is indicated when information regarding the orchard size (expressed in ha) was not available.

**Supplementary Table S2.** Results of one-way ANOVA testing the effects of different time periods (P1-P4), geographical areas (Gebele, Khachmaz and Zaqatala) and years (2023-2025), on selected meteorological variables (Tm, RHm and ΣDD).

| **Variable** | **Factor** | **F value** | **P value** | **Significance** |
| --- | --- | --- | --- | --- |
| Tm | Period | 1221.0 | < 0.001 | ** |
|  | Area | 0.046 | 0.955 | n.s |
|  | Year | 0.005 | 0.995 | n.s |
| RHm | Period | 7.817 | < 0.001 | ** |
|  | Area | 8.528 | 0.001 | ** |
|  | Year | 0.085 | 0.919 | n.s |
| ΣDD | Period | 778.8 | < 0.001 | ** |
|  | Area | 0.183 | 0.834 | n.s |
|  | Year | 0.029 | 0.972 | n.s |

Notes**:**

** *P* < 0.01; n.s. = not significant.

**Supplementary Table S3.** Log_10_(CFU/g) values of major fungal genera isolated from late-harvest hazelnut samples collected in September 2025 across orchards in the Zaqatala region (Azerbaijan). Each orchard is represented by two paired samples, one collected directly from the tree and one from the ground. Quantified fungal groups include *Alternaria spp.*, *Aspergillus* section *Flavi* and *Nigri*, *Fusarium spp.*, and *Penicillium spp.*

| **Sample Code** | **Collection Date** | **Collection Point** | ***A.* section *Flavi*** | ***A.* section *Nigri*** | ***Alternaria spp.*** | ***Fusarium spp.*** | ***Penicilium spp.*** |
| --- | --- | --- | --- | --- | --- | --- | --- |
| ZRC n39 | 06-Sept | Ground | 4.2 | 2.8 | 2.7 | 3.0 | 3.6 |
| ZRC n39 | 06-Sept | Tree | 3.6 | 3.3 | 0.0 | 5.2 | 3.8 |
| Zaqatala 1 | 10-Sept | Ground | 4.2 | 3.6 | 0.0 | 2.6 | 3.4 |
| Zaqatala 1 | 10-Sept | Tree | 4.7 | 4.5 | 0.0 | 0.0 | 2.9 |
| Zaqatala 2 | 11-Sept | Ground | 3.8 | 2.9 | 0.0 | 3.3 | 3.0 |
| Zaqatala 2 | 11-Sept | Tree | 3.5 | 3.3 | 0.0 | 3.6 | 4.3 |
